# Liposomal Delivery of *Allolobophora caliginosa* Coelomic Fluid Attenuates Myocardial Infarction by Suppressing Oxidative Damage, Inflammation, and Apoptosis

**DOI:** 10.1007/s12010-025-05340-y

**Published:** 2025-07-16

**Authors:** Asmaa E. Farouk, Sohair R. Fahmy, Amel M. Soliman, Sherif Abdelaziz Ibrahim, Shimaa A. Sadek

**Affiliations:** Zoology Department, Faculty of Science, Cairo University, Giza, 12613 Egypt

**Keywords:** Myocardial infarction, Adrenaline, *Allolobophora caliginosa*, Coelomic fluid, Liposomes, Oxidative stress, DNA fragmentation

## Abstract

Myocardial infarction (MI) is a concerning coronary heart disease with increasing rates of death and morbidity worldwide. One potential approach to prevent MI involves exploring invertebrate supplements within the nanoliposome formulation to improve targeted delivery, thereby mitigating MI-induced heart damage. Therefore, the study aimed to evaluate the cardioprotective efficacy of liposomal delivery of *Allolobophora caliginosa* coelomic fluid (ACCF-liposomes) on adrenaline-induced MI in rats. Thirty male albino rats were allocated into five groups: Control, Untreated MI, MI-treated ACCF, MI-treated free liposomes, and MI-treated ACCF-liposomes. The treatment regimen spanned 21 days. Electrocardiography (ECG), biochemical, oxidative stress, inflammatory mediators, electrolyte balance, histopathological and immunohistochemical analyses, and DNA fragmentation were evaluated. Liposomal delivery of ACCF has shown promise in regulating ECG criteria and reducing myocardial markers, particularly AST, LDH, MMP-2, creatine kinase, and troponin-I. It also improves lipid metabolism and inhibits myocardial oxidative stress. Additionally, ACCF and ACCF-liposomes treatment improves cardiomyocyte architecture and reduces DNA fragmentation in myocardial infarcted rats. Furthermore, encapsulating ACCF within liposomes statistically reduced the expression of iNOS and Beclin-1 in cardiac tissue. This suggests that liposomal delivery of ACCF enhances its effectiveness in treating myocardial infarction, potentially via its antioxidant, anti-inflammatory, and anti-apoptotic attributes.

## Introduction

Cardiovascular diseases (CVD) are the leading cause of global mortality, resulting in approximately 20.5 million deaths in 2021, and accounting for one-third of all global deaths [1]. Among critical cardiovascular diseases, myocardial infarction (MI) accounts for approximately 76.5% of sudden cardiac deaths and has become a significant public health concern [2]. Myocardial infarction arises from an insufficient delivery of blood to the myocardium relative to its demand, resulting in the necrosis of cardiomyocytes due to prolonged myocardial ischemia [3]. Notably, the prevalence and fatality rates of MI have shown a marked increase in Egypt in recent decades compared to other nations within the region and globally [4]. This condition demands prompt attention and strategic intervention to minimize its harmful impact.

Multiple pathogenic mechanisms are implicated in myocardial infarction (MI); however, the imbalance between myocardial oxidants and antioxidants significantly contributes to the progression of MI [5]. Ischemic tissue releases reactive oxygen species (ROS), damaging cellular structures, mitochondrial dysfunction, inflammatory and necrotic processes, and initiating apoptotic signaling cascades. These cascades ultimately lead to cardiac dysfunction and myocardial cell death [6]. In addition, inflammation plays a role in endothelial dysfunction, foam cell formation, plaque development and progression, and eventual rupture. It also contributes to weakening plaques’ fibrous caps, becoming a significant factor in myocardial infarction [7].

Minimizing ischemic time using reperfusion therapies, such as thrombolytic therapy or primary percutaneous coronary intervention, has effectively reduced morbidity and mortality associated with MI [8]. This is achieved by mitigating excessive clot formation and reducing the likelihood of recurrent coronary artery obstructions [9]. However, the use of these medications presents challenges due to their adverse side effects. Consequently, ongoing scientific research explores biologically active components in natural products with potential cardiovascular benefits, particularly those found in earthworms [10]. The earthworm species *Allolobophora caliginosa* is known for its various medicinal properties, including antiulcer, anticoagulant, antiviral, antibacterial, antifungal, antitumor, anti-inflammatory, cytotoxic, antipyretic, and analgesic effects [11, 12]. Notably, these effects are attributed to its coelomic fluid, which contains antioxidants, anti-inflammatory compounds, and fibrinolytic enzymes [13]. Moreover, coelomocytes suspended and circulating in the coelomic fluid have been shown to play a role in clot formation, functioning similarly to vertebrates’ thrombocytes [14]. Although coelomic fluid offers promising cardioprotective benefits, its clinical application may be hindered by limited intestinal absorption and multidrug resistance, resulting in reduced bioavailability [15].

A recent trend in natural product-based drug discovery involves utilizing new drug delivery systems to target specific body parts precisely. This innovative approach is expected to address the challenges of drug accumulation, reduce overall drug dosage, and concurrently enhance bioavailability [16]. Significantly, liposome formulation-based drug delivery has gained considerable attention in developing new pharmaceutical nanocarriers and delivery systems to overcome natural agents’ physicochemical and pharmacokinetic limitations [17]. Liposomes are phospholipid vesicles comprising one or more concentric lipid bilayers that enclose discrete aqueous spaces. These vesicles can entrap lipophilic and hydrophilic compounds and are proficient in encapsulating substantial quantities of drugs while ensuring controlled release of the encapsulated contents [18]. Moreover, liposomes exhibit distinctive physical characteristics, encompassing small particle size, charge, lipid composition, and surface modification with polymers, which enhance their stability in vitro* and *in vivo [19]*.* These characteristics allow liposomes to passively target inflammatory sites and facilitate drug release upon uptake by local macrophages, rendering them a promising drug delivery system for myocardial infarction treatment [20]. Schriefl et al. [21] discovered that the heightened vascular permeability after a cardiac arrest enables nanocarriers like liposomes to accumulate in the affected cardiac tissue. This suggests that liposomes could be a potential drug delivery system for the earthworm coelomic fluid, enhancing its bioavailability. Our previous research demonstrates the successful development of ACCF-liposomes characterized by a uniform size distribution, with an average diameter of approximately 98 nm and a polydispersity index (PDI) of 0.29 ± 0.04, indicating good stability [22]. When ACCF was encapsulated within the liposomes, the surface charge became negative (− 38.66 ± 0.33 mV), facilitating stability in biological environments. Furthermore, the entrapment efficiency was determined to be 77.58 ± 0.82%, confirming the effective loading of ACCF [22]. Furthermore, our research team demonstrated that liposomes could improve the stability, release behavior, and biological activities of ACCF, encompassing antioxidant and anti-inflammatory effects, as well as fibrinolytic potency. These attributes reinforce the potential of liposomes as stable carriers for ACCF, enabling controlled release and enhanced bioavailability in the intended application. So, the current study aims to assess the cardioprotective efficacy of *Allolobophora caliginosa* coelomic fluid (ACCF) and its liposomal formulation (ACCF-liposomes) on adrenaline-induced myocardial infarction in male albino rats.

## Materials and Methods

### Chemicals and Reagents

Alpha Chemical (Mumbai, India) provided the phospholipid component of the liposomes (soybean lecithin, 98.7% purity). Sigma Aldrich (St. Louis, Missouri, USA) acquired cholesterol. Adrenaline ampoules (1 mg/mL) were purchased from a local pharmacy (Dokki, Giza, Egypt). All reagents utilized in the experiment were of analytical grade.

### Collection of *Allolobophora caliginosa* Coelomic Fluid (ACCF)

Coelomic fluid was collected directly from the earthworms’ body cavity using the heat shock method described by Dinesh et al. [23]. In a controlled environment, 3–4 healthy earthworms were carefully positioned within a sterile Petri dish and subjected to a heat shock treatment using hot water at 45–50 °C. After exposure to heat shock, the coelomic fluid was released through the dorsal epidermal pore into the media and collected at the bottom of the Petri plate. Afterward, the coelomic fluid was centrifuged at 4000 rpm for 30 min at 4 °C. Finally, the resultant supernatant was concentrated and dried using a lyophilizer (EDWARDS, Italy).

### Preparation of ACCF-liposomes

The ACCF liposomal formulation was prepared using the lipid film hydration method, as Chen et al. [24] described. Briefly, 225 mg of soy lecithin and 25 mg of cholesterol were dissolved in 6 mL dichloromethane. Forty-five milligrams of ACCF was added, evaporating the mixture to form a dry lipid film. The film was then hydrated with a phosphate-buffered saline solution and sonicated for 5 min at a measured power of 120 W in an ice bath before being stored at 4 °C. The free liposomes were prepared similarly without incorporating ACCF. Finally, the prepared liposomes were concentrated and dried using a lyophilizer (EDWARDS, Italy). Our research team previously analyzed the physicochemical characterization of the resulting ACCF-liposomes [22].

### Experimental Animals

Adult 8-week-old Wistar rats weighing 150–180 g were supplied from the National Research Centre (NRC) in Egypt. They were housed in polypropylene cages with four animals per cage and acclimatized for seven days. They were provided with standard chow pellets and had access to drinking water ad libitum. Experimental protocols were approved by Cairo University’s Institutional Animal Care and Use Committee (IACUC) (Egypt) (Approval No. CU/I/F/58/21), adhering to international guidelines for the care and use of laboratory animals.

### Cardioprotection Protocol and Treatment Schedule

**Fig. 1:**
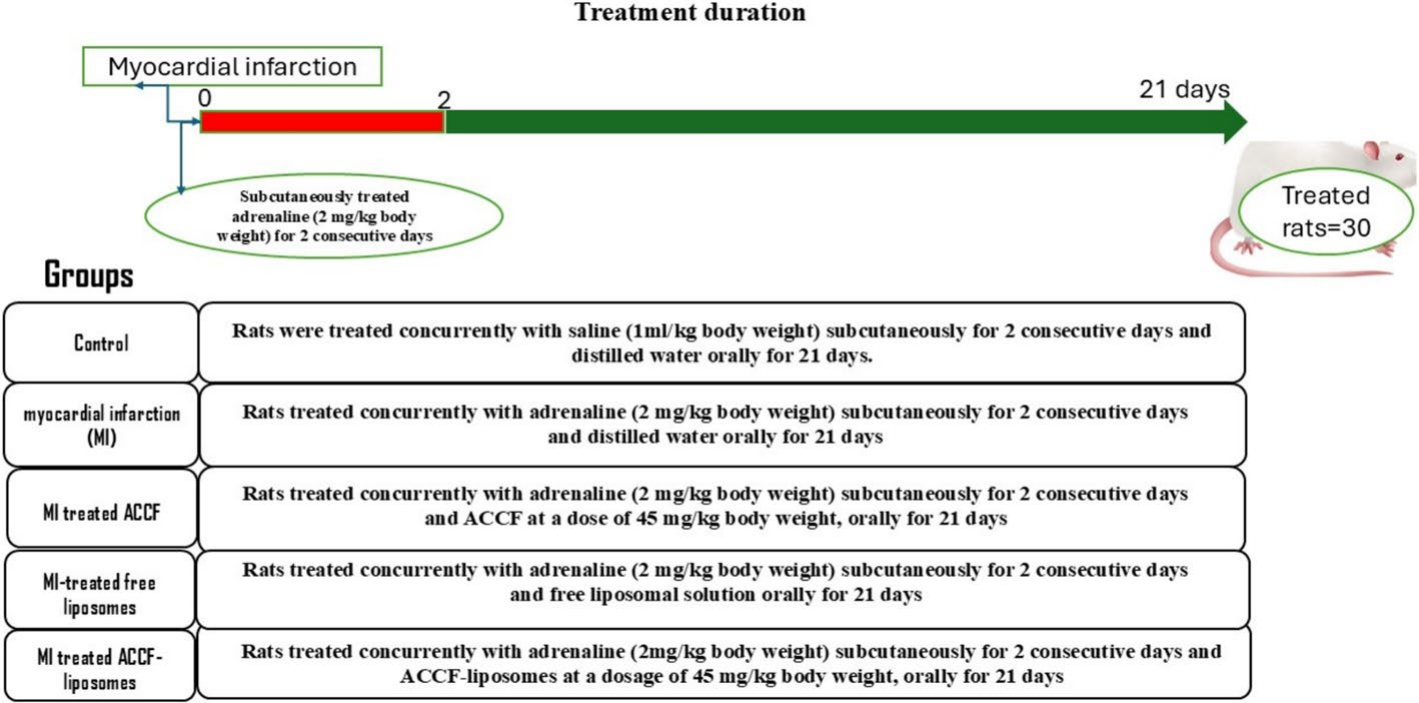
Diagram illustrates the cardioprotection protocol and treatment schedule of different experimental groups

Thirty healthy adult rats were randomly allocated into five groups (6 rats/group), as illustrated in Fig. 1.

- Group I: Control; rats were administered saline subcutaneously for two consecutive days and distilled water orally for 21 days concurrently.
- Group II: myocardial infarction (MI); rats were administered concurrently with adrenaline (2 mg/kg body weight) subcutaneously for two consecutive days and distilled water orally for 21 days.
- Group III: MI-treated ACCF; rats were administered adrenaline (2 mg/kg body weight) subcutaneously for two consecutive days, along with ACCF at a dose of 45 mg/kg body weight, administered orally for 21 days.
- Group IV: MI-treated free liposomes; rats were administered concurrently with adrenaline (2 mg/kg body weight) subcutaneously for two consecutive days, along with oral administration of free liposomal solution for 21 days.
- Group V: MI-treated ACCF-liposomes; rats were concurrently administered subcutaneous adrenaline at a dosage of 2 mg/kg body weight for two consecutive days, along with oral administration of ACCF-liposomes at a dosage of 45 mg/kg body weight for 21 days.

The doses of ACCF were chosen based on previously published data by Dajem et al. [25].

### Electrocardiogram (ECG) Assessment

On the 22^nd^ day, rats were anesthetized with sodium pentobarbital (50 mg/kg body weight) and placed supine in all groups. The ECG was recorded for 1 min using subcutaneous peripheral limb electrodes and an ECG PowerLab module, which includes a PowerLab/8SP and an animal bio-amplifier (Australia). LabChart 7.3.8 software with an ECG analyzer was used to record and analyze heart rate (HR), R-R interval, QRS interval, R amplitude, and P wave duration.

### Animal Handling and Specimen Assembly

At the end of the experiment, all overnight fasted rats were euthanized under anesthesia with sodium pentobarbital (50 mg/kg body weight). Then, cardiac puncture immediately collected blood into centrifuge tubes, and heart samples were quickly excised and weighed. After that, the heart was instantly cleaned with physiological saline to eliminate blood traces, and the left ventricle was dissected and weighed to determine heart hypertrophy indices. The left ventricle and other parts of the heart were divided into three portions: one for oxidative stress assays, another for histopathological and immunohistochemistry examinations, and the third for DNA fragmentation assay.

### Measurement of Hypertrophy Indices

The rats’ body weight was recorded before euthanasia. Then, the left ventricles were weighed after euthanasia to determine the ratios of heart weight to body weight and left ventricular weight to heart weight. These ratios were identified as heart hypertrophy indices.

### Determination of Cardiac Function Markers

Serum cardiac-specific troponin-I level (cTn-I) and matrix metalloproteinase 2 (MMP-2) levels were analyzed using the enzyme-linked immunosorbent assay (ELISA) technique with specific kits for rat specimens. Additionally, creatine kinase (CK-MB) was assessed using the in vitro endpoint method according to the method described by Guder et al. [26]. Aspartate aminotransferase (AST) and lactate dehydrogenase (LDH) activity were assessed using a Vitro kit [27], and the uric acid level was measured using a Biodiagnostic kit based on the colorimetric method [28].

### Assessment of Lipid Profile and Cardiovascular Risk Indices

The Vitro kit with the enzymatic colorimetric method I (CHOD/PAP) measured the serum total cholesterol and HDL cholesterol levels. Triglycerides were determined using a Biodiagnostic kit, and the concentration of LDL cholesterol was determined using a Spectrum kit. Subsequently, a series of cardiovascular risk indices were calculated, which included Castelli’s Risk Index I (CRI-I), Castelli’s Risk Index II (CRI-II), atherogenic coefficient (AC), non–high–density lipoprotein (NHC), and atherogenic index of plasma (AIP), using specific equations [29].

### Evaluation of Electrolyte Balance

Serum potassium, sodium, calcium, and total phosphorus levels were determined colorimetrically using the in vitro endpoint method, according to Hoeflmayr [30].

### Determination of Cardiac Oxidative and Antioxidative Markers

The heart tissues were homogenized in 0.1 M Tris–HCl buffer (pH = 7.4) to yield a 10% homogenate at 4 °C. Subsequently, the homogenate samples underwent centrifugation at 3000 rpm for 15 min, and the resulting supernatant was utilized to assess oxidative stress and antioxidant parameters. The acquired heart supernatant was employed for the determination of malondialdehyde (MDA), nitric oxide (NO), glutathione reduced (GSH), superoxide dismutase (SOD), glutathione peroxidase (GPx), and glutathione-S-transferase (GST) using commercially available kits.

### Detection of DNA Fragmentation by Using Ladder Assay

DNA was extracted utilizing the DMSO (dimethyl sulfoxide)–SDS (sodium dodecyl sulfate)–TE (Tris–EDTA) method [31]. Cardiac tissues underwent a double wash with PBS (phosphate-buffered saline, pH 7.4) and were subsequently lysed in 500 µL of DMSO. An equivalent volume of Tris EDTA buffer (500 μL, pH 7.4) with 2% SDS was introduced, followed by vertexing of the samples. The resultant solution was then centrifuged at 12,000 rpm at 4 °C, and 40 µL of the supernatant was processed and separated on a 2% agarose gel at 60 V. The fragmented DNA was visualized using the BioSpectrum 815 Imaging System (UVP, CA, USA).

### Histopathological Study

Heart tissues were rinsed in saline and then fixed in neutral buffered formalin (10%, pH 7.0) for 24 h. Subsequently, the tissues underwent standard processing methods to obtain paraffin sections, which were then cut and stained with hematoxylin–eosin (H&E) for examination.

### Immunohistochemical Study of Beclin-1 and iNOS Expression

The heart sections were immunostained to detect Beclin-1 (Bcl-1) and iNOS using the streptavidin-biotinylated horseradish peroxidase method. Histological analysis was conducted on 4-μm-thick longitudinal heart sections using the following protocol: the sections were deparaffinized in xylene, rehydrated in ethanol, and subjected to microwave-assisted antigen retrieval. Subsequently, overnight incubation with antibodies at 4 °C was performed, followed by visualization of the bound complexes and counterstaining with hematoxylin. Negative control slides were prepared from the heart following standardized procedures; however, they were treated with antibody diluent instead of the primary antibody during incubation.

### Morphometric Study

The analysis utilized ImageJ software to examine 10 non-overlapping fields for each section. The area percentage of Beclin-1 and iNOS immunoreactivity was quantified at a magnification of 40 × under a light microscope.

### Statistical Analysis

All biochemical data were expressed as mean ± standard error. Group comparisons were assessed using one-way analysis of variance (ANOVA), followed by the Duncan post hoc test using SPSS software (SPSS Inc., Chicago, IL, USA). For the morphometric study, a one-way ANOVA was followed by a Tukey post hoc test using GraphPad Prism software. Statistically significant values were defined as *P* < 0.05. Lastly, the efficacy of ACCF and its nanoliposomal formulation was evaluated by calculating the percentage of change compared to the MI group.

## Results

### Effect of ACCF and ACCF-liposomes on Body Weight Change and Hypertrophy Indices

Table 1 shows that administering adrenaline at a dose of 2 mg/kg body weight for two consecutive days resulted in myocardial infarction. This was evidenced by a significant (*P* < 0.05) increase in relative heart weight and ventricular weight ratio, along with a noticeable decrease in body weight compared to the control group. On the other hand, myocardial infarcted rats treated with either free ACCF or its liposomal formulation orally at a dose of 45 mg/kg body weight for 21 days exhibited a significant (*P* < 0.05) improvement in their relative heart weight, ventricular weight ratio, and body weight gain compared to untreated rats with myocardial infarction. Furthermore, oral administration of the free liposomal formulation for 21 days led to a significant (*P* < 0.05) decrease in relative heart weight and ventricular weight ratio. However, the change in body weight after free liposome administration was not significantly different from the untreated myocardial infarction group.

**Table 1:** The cardioprotective potency of ACCF and ACCF-liposomes on body weight change and hypertrophy indices of myocardial infarcted rats

Experimental groups	Body weight change (g)	Relative heart weight (g/100 g)	Ventricular weight ratio (%)
Control	1.055 ± 0.11^c^	0.36 ± 0.02^a^	3.44 ± 0.56^a^
MI	0.56 ± 0.02^a^	0.78 ± 0.02^d^	45.41 ± 4.76^d^
MI + ACCF	0.66 ± 0.01^b^	0.53 ± 0.01^b^	17.92 ± 0.69^b^
MI + free liposomes	0.70 ± 0.09^a^	0.65 ± 0.02^c^	35.07 ± 1.82^c^
MI + ACCF-liposomes	0.97 ± 0.02^c^	0.41 ± 0.02^a^	6.16 ± 0.63^a^

Values are expressed as mean ± SEM (*n* = 6)

Values with different superscript letters are significantly different (*P* < 0.05)

*MI* myocardial infarction, *ACCF Allolobophora caliginosa* coelomic fluid, *ACCF-liposomes* liposomes entrapped *Allolobophora caliginosa* coelomic fluid

### Effect of ACCF and ACCF-liposomes on ECG Changes

**Fig. 2:**
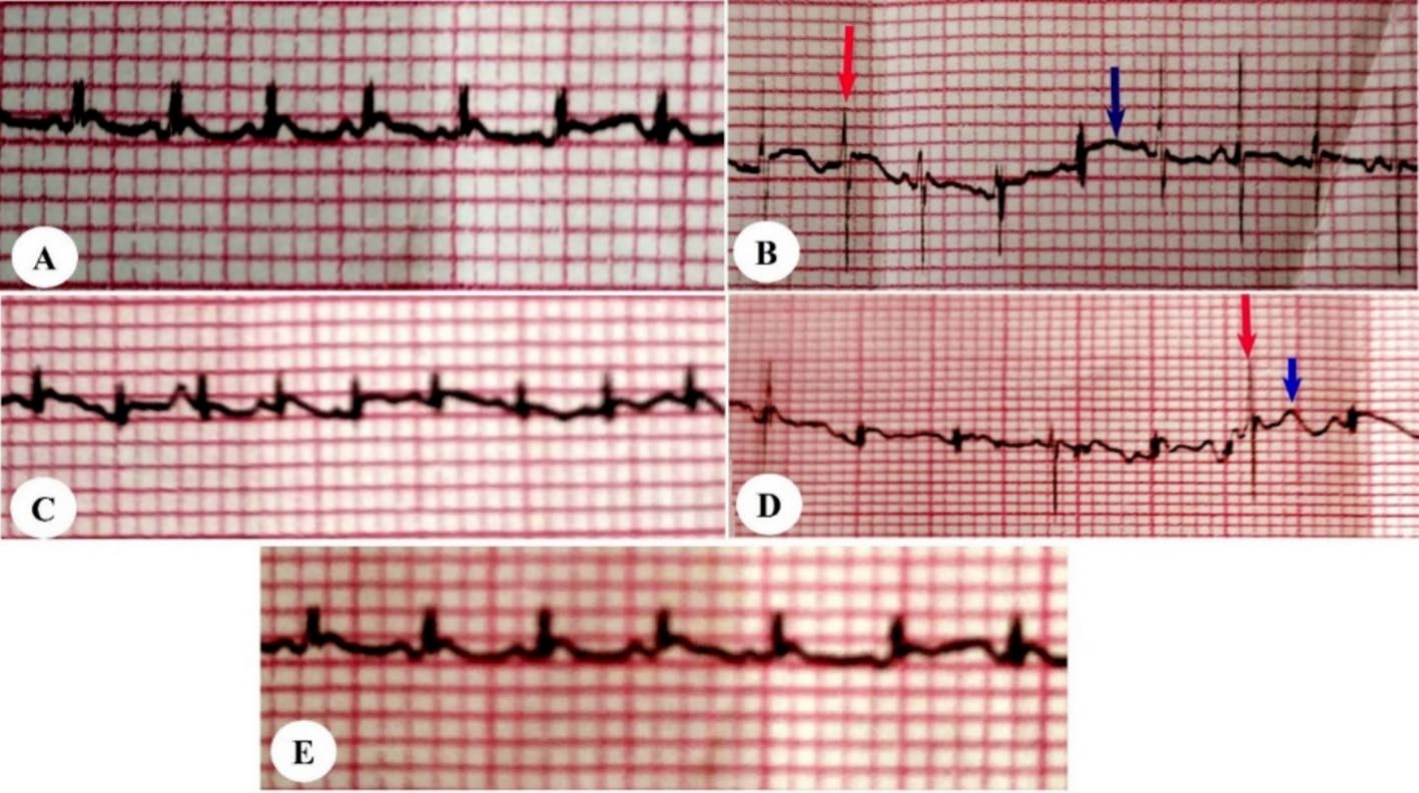
Cardioprotective efficacy of ACCF and ACCF-liposomes on ECG changes caused by adrenaline. **A** Control group. **B** Untreated myocardial infarcted group. **C** Myocardial infarcted group treated with ACCF (45 mg/kg body weight). **D** Myocardial infarcted group treated with free liposomes. **E** Myocardial infarcted group treated with ACCF-liposomes (45 mg/kg body weight). The red arrow indicates pathologic QRS waves; the blue arrow indicates ST-segment elevation

As shown in Fig. 2A, the control group displayed typical ECG patterns, including a normal P wave followed by a QRS complex and a T wave. Figure 2B shows that subcutaneous administration of adrenaline (2 mg/kg body weight) resulted in pathological changes in ECG patterns compared to the control group. These changes included ST-segment elevation, negative T waves, and abnormal QRS waves. Interestingly, these abnormalities were improved with the treatment of ACCF or its liposomal formulation at 45 mg/kg body weight (Fig. 2C, E). Additionally, myocardial infarcted rats treated with the free liposomal formulation did not show improvement in ECG changes caused by adrenaline administration (Fig. 2D).

### Effect of ACCF and ACCF-liposomes on ECG Criteria

Rats administered subcutaneous adrenaline injection at a dosage of 2 mg/kg of body weight for two consecutive days demonstrated a statistically significant (*P* < 0.05) increase in their R-R interval, P-wave duration, QRS interval, and ST-segment while manifesting a reduction in heart rate and R-wave amplitude (Table 2). Conversely, myocardial infarcted rats treated with ACCF or its liposomal formulation at a dosage of 45 mg/kg of body weight for 21 days exhibited a marked improvement in their ECG parameters compared to untreated myocardial infarcted rats. Notably, the R-R interval, P-wave duration, QRS interval, heart rate, and R-wave amplitude of myocardial infarcted rats depicted insignificant alterations after administering the free liposomal formulation. However, their ST segment exhibited a statistically significant (*P* < 0.05) increase compared to the untreated MI group (Table 2).

**Table 2:** The cardioprotective potency of ACCF and ACCF-liposomes on ECG criteria of myocardial infarction rats

Experimental groups	R-R interval (ms)	P wave duration (ms)	QRS interval (ms)	ST segment (mv)	Heart rate (beat/min)	R wave amplitude (mv)
Control	18.67 ± 0.59^a^	36.00 ± 3.27^a^	120.00 ± 10.32^a^	0.04 ± 0.01^a^	370.00 ± 29.86^b^	0.86 ± 0.04^c^
MI	27.89 ± 1.04^b^	64.00 ± 8.01^b^	380.00 ± 48.98^b^	0.12 ± 0.01^b^	222.73 ± 9.81^a^	0.45 ± 0.05^a^
MI + ACCF	17.50 ± 0.61^a^	40.00 ± 5.16^a^	180.00 ± 21.91^a^	0.07 ± 0.01^a^	387.52 ± 26.22^b^	0.68 ± 0.03^b^
MI + free liposomes	28.00 ± 1.46^b^	65.00 ± 6.06^b^	340.00 ± 28.76^b^	0.17 ± 0.03^c^	239.00 ± 5.37^a^	0.40 ± 0.03^a^
MI + ACCF-liposomes	16.80 ± 1.22^a^	30.00 ± 3.65^a^	96.50 ± 8.67^a^	0.03 ± 0.01^a^	370.00 ± 29.86^b^	0.73 ± 0.03^b^

Values are expressed as mean ± SEM (*n* = 6)

Values with different superscript letters are significantly different (*P* < 0.05)

*MI* myocardial infarction, *ACCF Allolobophora caliginosa* coelomic fluid, *ACCF-liposomes* liposomes entrapped *Allolobophora caliginosa* coelomic fluid

### Effect of ACCF and ACCF-liposomes on Cardiac Function Markers

The results presented in Table 3 indicate a significant (*P* < 0.05) increase in serum cardiac function markers in rats following subcutaneous administration of adrenaline at a dosage of 2 mg/kg body weight for two consecutive days, compared to the control group. Moreover, myocardial infarcted rats treated with ACCF and ACCF-liposomes for 21 days at a dosage of 45 mg/kg body weight exhibited a noteworthy reduction in serum CK-MB, AST, LDH activities, cTn-I level, and uric acid concentration. Specifically, ACCF-liposomes treatment led to a significant (*P* < 0.05) decrease in MMP-2 activity compared to untreated myocardial infarcted rats. Notably, the free liposomal formulation administration for 21 days resulted in a non-significant change in serum MMP-2, CK-MB, and cTn-I levels compared to the untreated MI group. However, compared to the untreated MI group, the levels of AST, LDH, and uric acid in myocardial infarcted rats significantly decreased after the free liposomal formulation treatment.

**Table 3:** The cardioprotective potency of ACCF and ACCF-liposomes on cardiac markers of myocardial infarcted rats

Experimental groups	CK-MB (U/L)	cTnI (ng/mL)	MMP 2 (ng/mL)	AST (U/L)	LDH (U/L)	Uric acid (mg/dL)
Control	27.03 ± 1.48^a^	0.29 ± 0.04^a^	11.79 ± 1.42^a^	20.83 ± 2.93^a^	80.22 ± 15.13^a^	2.58 ± 0.09^a^
MI	63.92 ± 8.15^b^	0.81 ± 0.05^c^	17.45 ± 0.78^b^	111.42 ± 11.02^d^	375.99 ± 32.49^c^	3.93 ± 0.23^c^
MI + ACCF	15.60 ± 1.09^a^	0.51 ± 0.04^b^	15.03 ± 0.40^b^	29.63 ± 6.29^ab^	55.98 ± 6.95^a^	2.55 ± 0.22^a^
MI + free liposomes	63.10 ± 5.90^b^	0.87 ± 0.04^c^	16.53 ± 0.36^b^	88.17 ± 9.69^c^	149.68 ± 17.99^b^	3.19 ± 0.13^b^
MI + ACCF-liposomes	21.50 ± 2.60^a^	0.48 ± 0.06^b^	12.01 ± 0.68^a^	45.00 ± 5.45^b^	55.99 ± 3.64^a^	2.09 ± 0.17^a^

Values are expressed as mean ± SEM (*n* = 6)

Values with different superscript letters are significantly different (*P* < 0.05)

*MI* Myocardial infarction, *ACCF Allolobophora caliginosa* coelomic fluid, *ACCF-liposomes* liposomes entrapped *Allolobophora caliginosa* coelomic fluid

### Effect of ACCF and ACCF-liposomes on Lipid Profile

The subcutaneous administration of adrenaline at a dosage of 2 mg/kg body weight for two consecutive days resulted in a statistically significant (*P* < 0.05) increase in serum total cholesterol, triglycerides, and LDL-cholesterol levels, while there was a statistically significant (*P* < 0.05) decrease in HDL-cholesterol levels when compared to the control group (Table 4). Moreover, in myocardial infarcted rats, oral administration of ACCF and ACCF-liposomes at a dosage of 45 mg/kg body weight for 21 days led to a significant (*P* < 0.05) decrease in serum total cholesterol, triglycerides, and LDL-cholesterol levels, and a significant (*P* < 0.05) increase in HDL-cholesterol levels when compared to untreated myocardial infarcted rats. In contrast, no substantial changes in the total cholesterol, triglyceride, LDL-cholesterol, and HDL-cholesterol levels were observed in myocardial infarcted rats following the free liposomal formulation administration compared to the myocardial infarcted group.

**Table 4:** The cardioprotective potency of ACCF and ACCF-liposomes on lipid profile of myocardial infarcted rats

Experimental groups	Total cholesterol (mg/dL)	Triglycerides (mg/dL)	LDL-cholesterol (mg/dL)	HDL-cholesterol (mg/dL)
Control	45.09 ± 1.69^a^	331.40 ± 15.74^a^	383.41 ± 31.66^a^	570.23 ± 15.95^b^
MI	66.77 ± 3.00^c^	457.59 ± 14.49^b^	725.14 ± 29.14^c^	491.82 ± 19.64^a^
MI + ACCF	55.46 ± 0.69^b^	348.78 ± 10.67^a^	545.67 ± 12.95^b^	550.65 ± 21.54^ab^
MI + free liposomes	63.09 ± 2.43^c^	490.18 ± 23.18^b^	690.80 ± 27.82^c^	433. 60 ± 30.24^a^
MI + ACCF-liposomes	50.69 ± 1.79^ab^	336.81 ± 17.22^a^	499.02 ± 26.22^b^	618.87 ± 51.09^b^

Values are expressed as mean ± SEM (*n* = 6)

Values with different superscript letters are significantly different (*P* < 0.05)

*MI* myocardial infarction, *ACCF Allolobophora caliginosa* coelomic fluid, *ACCF-liposomes* liposomes entrapped *Allolobophora caliginosa* coelomic fluid

### Effect of ACCF and ACCF-liposomes on Cardiovascular Risk Indices

In Table 5, it was found that rats administered adrenaline at a dose of 2 mg/kg body weight for two consecutive days showed a significant (*P* < 0.05) increase in their CRI-I, CRI-II, and AIP levels (*P* < 0.05), along with a marked decrease in their AC and NHC ratio compared to the control rats. On the other hand, rats with myocardial infarction, who were treated with either free ACCF or its liposomal formulation orally at a dose of 45 mg/kg body weight for 21 days, exhibited a significant (*P* < 0.05) decrease in their CRI-I, CRI-II, and AIP ratio (*P* < 0.05), while experiencing a marked increase in AC and NHC ratio compared to untreated myocardial infarcted rats. Moreover, the cardiovascular risk indices of myocardial infarcted rats did not significantly change after the administration of the free liposomal formulation compared to the untreated myocardial infarction group.

**Table 5:** The cardioprotective potency of ACCF and ACCF-liposomes on cardiovascular risk indices of myocardial infarcted rats

Experimental groups	CRI-I	CRI-II	AC	NHC	AIP
Control	0.09 ± 0.03^a^	0.67 ± 0.05^a^	0.92 ± 0.03^b^	552.93 ± 28.48^c^	0.58 ± 0.04^a^
MI	0.14 ± 0.06^c^	1.48 ± 0.05^c^	0.86 ± 0.06^a^	395.36 ± 41.23^a^	0.94 ± 0.05^c^
MI + ACCF	0.10 ± 0.04^b^	0.99 ± 0.03^b^	0.92 ± 0.02^b^	510.43 ± 26.68^b^	0.63 ± 0.02^b^
MI + free liposomes	0.15 ± 0.06^c^	1.57 ± 0.15^c^	0.87 ± 0.02^a^	404.14 ± 43.87^a^	1.05 ± 0.16^c^
MI + ACCF-liposomes	0.08 ± 0.01^a^	0.84 ± 0.09^a^	0.92 ± 0.08^b^	570.18 ± 51.84^c^	0.55 ± 0.04^a^

Values are expressed as mean ± SEM (*n* = 6)

Values with different superscript letters are significantly different (*P* < 0.05)

*MI* myocardial infarction, *ACCF Allolobophora caliginosa* coelomic fluid, *ACCF-liposomes* liposomes entrapped *Allolobophora caliginosa* coelomic fluid, *CRI-I* Castelli’s Risk Index I, *CRI-II* Castelli’s Risk Index II, *AC* atherogenic coefficient, *NHC* non–high–density lipoprotein, *AIP* atherogenic index of plasma

### Effect of ACCF and ACCF-liposomes on Serum Electrolytes

The subcutaneous administration of adrenaline at a dose of 2 mg/kg body weight over two days resulted in a statistically significant (*P* < 0.05) decrease in serum potassium levels and a concurrent increase in phosphorus, total calcium, and sodium levels in rats, as compared to the control group (Table 6). In contrast, the treatment with ACCF and ACCF-liposomes (45 mg/kg body weight) for 21 days led to a statistically significant (*P* < 0.05) rise in serum potassium levels and a decline in other electrolytes in myocardial infarcted rats, as compared to the untreated myocardial infarcted rats. Similarly, following the administration of the free liposomal formulation, a statistically significant (*P* < 0.05) increase in serum potassium levels and a significant (*P* < 0.05) decline in serum phosphorus levels were observed in myocardial infarcted rats relative to the untreated MI group. Nevertheless, the total calcium and sodium levels did not exhibit statistically significant changes after free liposomal administration relative to the untreated MI group.

**Table 6:** The cardioprotective potency of ACCF and ACCF-liposomes on serum electrolytes of myocardial infarcted rats

Experimental groups	Potassium (mmol/L)	Sodium (mEq/L)	Calcium (mg/dL)	Phosphorus (mg/dL)
Control	1.29 ± 0.07^c^	38.96 ± 4.25^a^	1.89 ± 0.07^b^	4.30 ± 0.28^a^
MI	0.86 ± 0.07^a^	81.82 ± 10.49^b^	3.39 ± 0.27^c^	8.61 ± 0.85^c^
MI + ACCF	1.66 ± 0.06^d^	36.91 ± 2.33^a^	1.18 ± 0.08^a^	4.10 ± 0.13^a^
MI + free liposomes	1.05 ± 0.02^b^	69.57 ± 9.27^b^	2.90 ± 0.32^c^	5.69 ± 0.18^b^
MI + ACCF-liposomes	1.62 ± 0.06^d^	41.85 ± 2.43^a^	1.28 ± 0.08^a^	3.68 ± 0.31^a^

Values are expressed as mean ± SEM (*n* = 6)

Values with different superscript letters are significantly different (*P* < 0.05)

*MI* myocardial infarction, *ACCF Allolobophora caliginosa* coelomic fluid, *ACCF-liposomes* liposomes entrapped *Allolobophora caliginosa* coelomic fluid

### Effect of ACCF and ACCF-liposomes on Cardiac Oxidative Markers

According to Table 7, the subcutaneous administration of adrenaline for two consecutive days resulted in a significant (*P* < 0.05) increase in myocardial MDA and NO content, as well as a significant (*P* < 0.05) decrease in GSH content in MI rats compared to the control group. Moreover, the treatment with both ACCF and ACCF-liposomes at a dosage of 45 mg/kg body weight for 21 days after myocardial infarction led to a noteworthy (*P* < 0.05) decrease in myocardial MDA level and NO content, along with an increase in GSH content compared to the untreated MI group. On the other hand, the levels of MDA, NO, and GSH in myocardial infarcted rats demonstrated insignificant changes after the administration of the free liposomal formulation compared to the untreated MI group.

**Table 7:** The cardioprotective potency of ACCF and ACCF-liposomes on cardiac oxidative stress markers of myocardial infarcted rats

Experimental groups	GSH (mg/g.tissue)	MDA (nmol/g.issue)	NO (μmol/L)
Control	0.25 ± 0.03^c^	0.39 ± 0.03^a^	22.26 ± 2.37^a^
MI	0.04 ± 0.01^a^	1.16 ± 0.07^b^	74.92 ± 4.83^b^
MI + ACCF	0.13 ± 0.02^b^	0.32 ± 0.03^a^	23.15 ± 4.83^a^
MI + free liposomes	0.05 ± 0.01^a^	1.04 ± 0.07^b^	68.87 ± 2.65^b^
MI + ACCF-liposomes	0.31 ± 0.01^c^	0.25 ± 0.06^a^	21.58 ± 4.30^a^

Values are expressed as mean ± SEM (*n* = 6)

Values with different superscript letters are significantly different (*P* < 0.05)

*MI* myocardial infarction, *ACCF Allolobophora caliginosa* coelomic fluid, *ACCF-liposomes* liposomes entrapped *Allolobophora caliginosa* coelomic fluid

### Effect of ACCF and ACCF-liposomes on Cardiac Antioxidant Enzyme Activities

The subcutaneous administration of adrenaline at a dose of 2 mg/kg body weight for two consecutive days resulted in a significant (*P* < 0.05) decrease in the activities of myocardial antioxidant enzymes (SOD, CAT, GPx, and GST) compared to the control group. However, treatment with ACCF and ACCF-liposomes (45 mg/kg body weight) for 21 days led to a significant (*P* < 0.05) increase in the activities of myocardial SOD, CAT, GPx, and GST in myocardial infarcted rats, compared to the untreated myocardial infarcted group (Table 8). Furthermore, myocardial infarcted rats treated with the free liposomal formulation for 21 days exhibited a non-significant change in their SOD, CAT, GPx, and GST activities relative to the untreated MI group (Table 8).

**Table 8:** The cardioprotective potency of ACCF and ACCF-liposomes on cardiac antioxidant enzyme activities of myocardial infarcted rats

Experimental groups	SOD (U/g.tissue)	(CAT) (U/g.tissue)	GPx (mU/mL)	GST (U/g.tissue)
Control	53.51 ± 7.24^c^	13.61 ± 2.17^c^	0.18 ± 0.06^b^	80.67 ± 10.68^b^
MI	7.56 ± 1.80^a^	2.86 ± 0.41^a^	0.11 ± 0.01^a^	23.16 ± 7.38^a^
MI + ACCF	32.33 ± 2.88^b^	7.51 ± 0.75^b^	0.18 ± 0.02^b^	98.47 ± 7.64^b^
MI + free liposomes	7.44 ± 1.51^a^	2.86 ± 0.29^a^	0.12 ± 0.05^a^	20.15 ± 2.28^a^
MI + ACCF-liposomes	35.39 ± 10.20^b^	18.14 ± 1.32^d^	0.19 ± 0.04^b^	174.36 ± 5.55^c^

Values are expressed as mean ± SEM (*n* = 6)

Values with different superscript letters are significantly different (*P* < 0.05)

*MI* myocardial infarction, *ACCF Allolobophora caliginosa* coelomic fluid, *ACCF-liposomes* liposomes entrapped *Allolobophora caliginosa* coelomic fluid

### Correlation Between Oxidative Stress Markers and Antioxidant Enzyme Levels

**Fig. 3:**
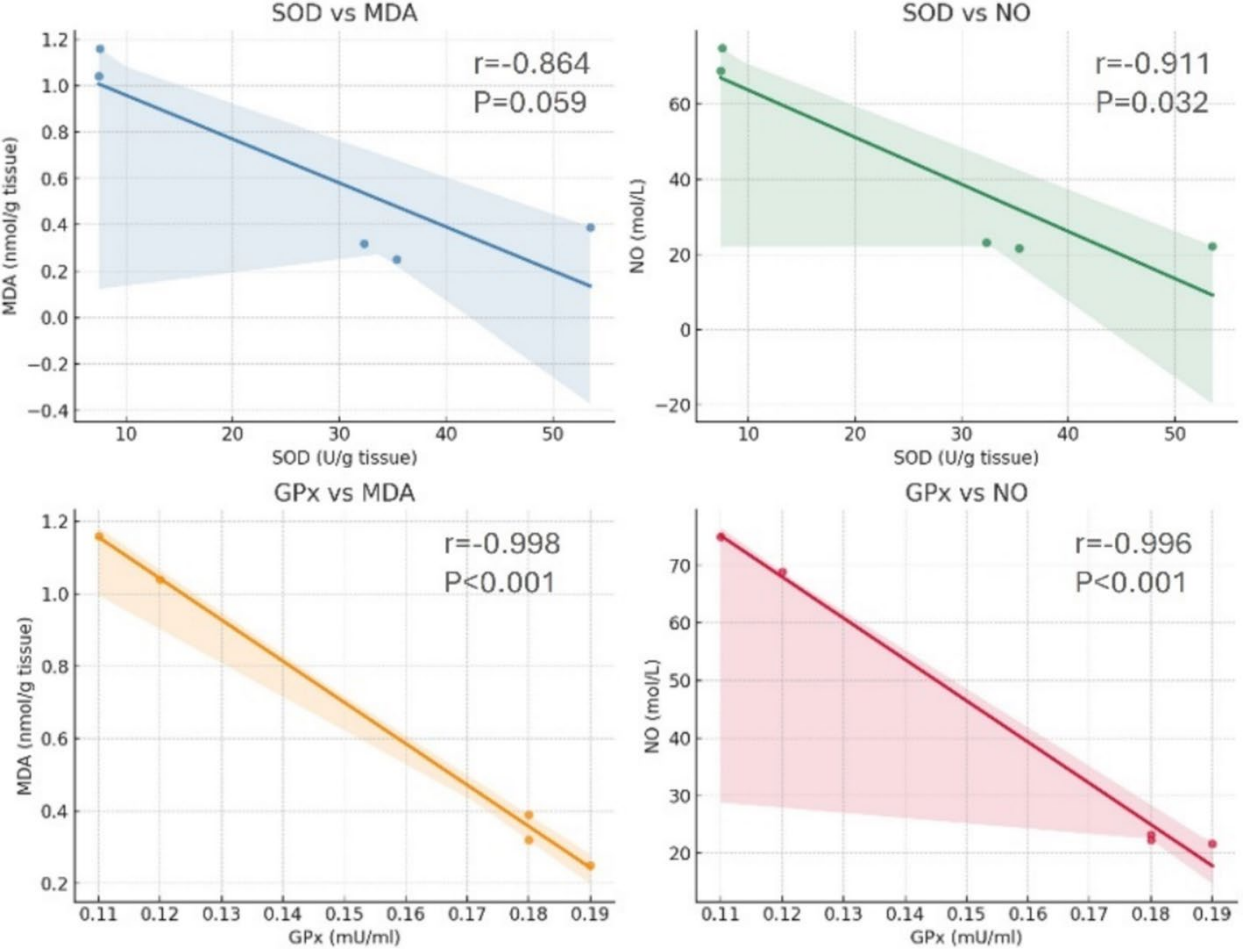
Pearson correlation between oxidative stress markers (MDA, NO) and antioxidant enzyme levels (SOD, GPx) in cardiac tissue

To elucidate the protective mechanism of ACCF-liposomes, a correlation analysis was performed between oxidative stress markers (MDA, NO) and antioxidant enzyme activities (SOD, GPx) across the experimental groups. As shown in Fig. 3, the results indicated a pronounced negative correlation between SOD and NO levels (*r* =  − 0.91, *P* = 0.032), implying that heightened SOD activity was linked to reduced nitric oxide levels. Additionally, SOD exhibited a significant inverse relationship with MDA (*r* =  − 0.86, *P* = 0.059), although this finding did not reach statistical significance. However, a strong inverse relationship was observed, particularly between GPx and both MDA (*r* =  − 0.998, *P* < 0.001) and NO (*r* =  − 0.996, *P* < 0.001), indicating that higher antioxidant activity is linked to lower oxidative damage.

### Effect of ACCF and ACCF-liposomes on DNA Fragmentation

**Fig. 4:**
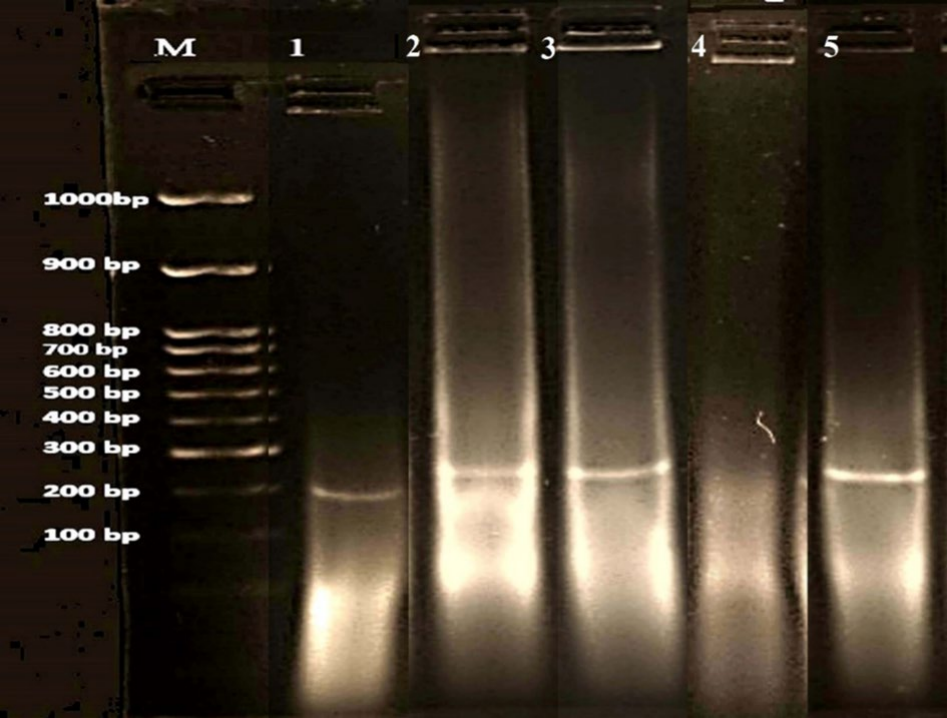
Agarose gel electrophoresis of DNA fragments extracted concerning the infarct size in different experimental groups. Lane 1: control group, Lane 2: myocardial infarction group, Lane 3: ACCF-treated group, Lane 4: free liposome-treated group, and Lane 5: ACCF-liposome-treated group

As shown in Fig. 4, agarose gel electrophoresis was conducted on DNA fragments extracted to assess the infarct size in different experimental groups. The degree of DNA fragmentation in myocardial tissue was more pronounced in untreated myocardial infarcted rats compared to the control group, indicating myocardial apoptosis. Meanwhile, no DNA fragmentation could be detected in myocardial cells treated with ACCF-liposomes, exhibiting a clear ladder pattern (Fig. 4, Lane 5). Slight fragmentation was observed in myocardial infarction treated with ACCF (Fig. 4, Lane 3).

### Effect of ACCF and ACCF-liposomes on Histological Changes of Myocardial Infarcted Rats

**Fig. 5:**
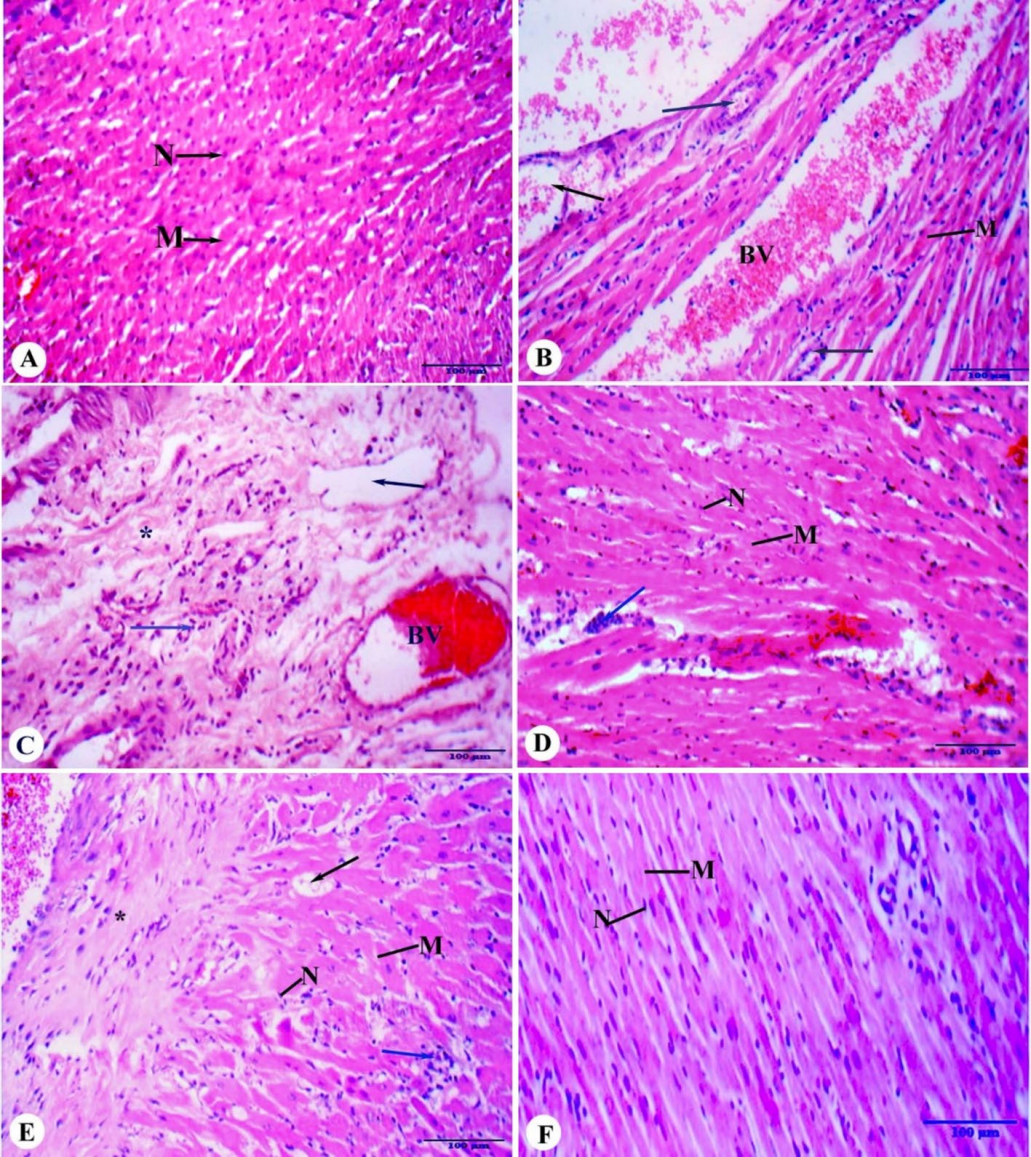
Photomicrograph of the rat’s myocardium architecture of different experimental groups stained with hematoxylin and eosin. **A** The control group showed well-preserved myocardial architecture with intact cardiac muscle fibers (M), central euchromatic oval nucleus (N), regular myofibrils arrangement, and acidophilic cytoplasm arrangement. **B **&** C** The myocardial infarction group showed severe damage to the heart muscle (indicated by an asterisk). This damage resulted in the destruction of muscle cells and the spaces around blood vessels, which were infiltrated by a mix of inflammatory cells, mostly eosinophils (blue arrow). The affected heart muscle cells looked paler than normal, with faint or soft sarcoplasm, vacuolation (black arrow), and congested blood vessels (BV). **D** The ACCF-treated group showed intact cardiac muscle fibers (M) with central nucleus (N) and few inflammatory cellular infiltrations (blue arrow). **E** The myocardial infarcted rats treated with free liposomes showed the destruction of muscle cells and space, which were infiltrated by a mix of inflammatory cells (blue arrow), congested capillaries in the cardiac muscle, and vacuolization (black arrow). **F** Myocardial infarcted rats treated with ACCF-liposomes showed nearly normal cardiac muscle fibers with central oval pale nuclei

In Fig. 5A, it was observed that the control group displayed a smooth surface with normal, well-preserved myocardial architecture. In comparison, the myocardial infarcted rats exhibited significant histological changes indicative of severe infarction progression, including severe myocardial necrosis, myocyte and perivascular space destruction, and a mixed inflammatory infiltrate mainly composed of eosinophils (Fig. 5B, C). The cardiomyocytes appeared paler with faint or soft sarcoplasm, vacuolation, and edema. Additionally, congested blood vessels were detected (Fig. 5B, C). Following a 21-day treatment with ACCF (45 mg/kg body weight), mild attenuation of the cardiac lesions was observed, with intact cardiac muscle fibers displaying central nuclei and a few inflammatory cellular infiltrations (Fig. 5D). Significant amelioration was noted in the ACCF-liposome-treated group (45 mg/kg body weight), with normal cardiomyocytes in most examined sections, nearly normal cardiac muscle fibers with central oval pale nuclei, and minimal amounts of collagen fibers noticed between cardiomyocytes in comparison with the control (Fig. 5F). Conversely, marked deterioration of cardiomyocyte necrosis and inflammatory cellular infiltrates was recorded in myocardial infarcted rats treated with the free liposome formulation for 21 days (Fig. 5E).

### Effect of ACCF and ACCF-liposomes on iNOS Expression of Myocardial Infarcted Rats

**Fig. 6:**
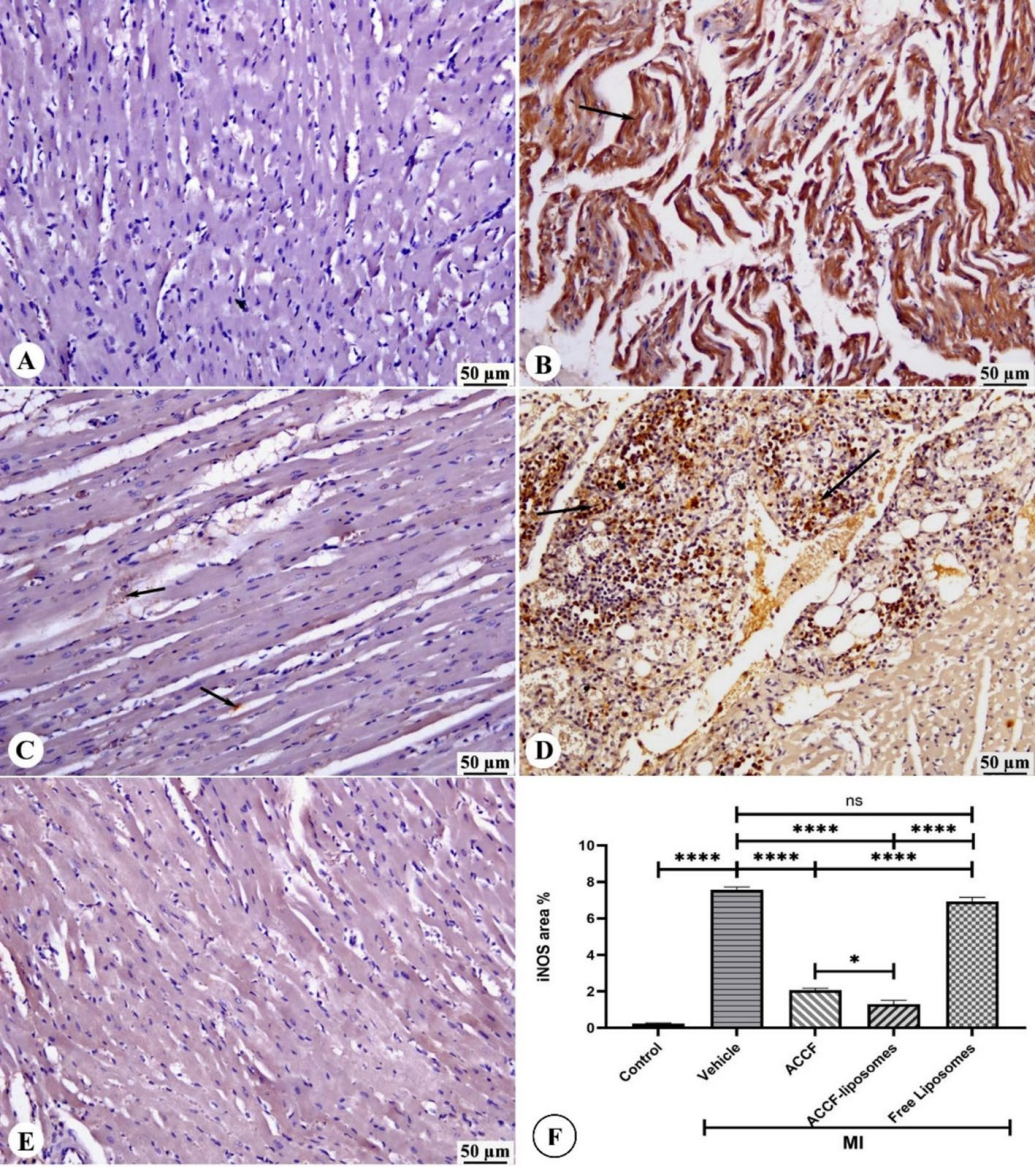
Photomicrograph of iNOS immunostained heart sections. **A** The control group shows limited iNOS expression in the cytoplasm of cardiomyocytes. **B** The myocardial infarcted group shows intense iNOS expression in the cytoplasm of cardiomyocytes (black arrow). **C** ACCF-treated group shows decreased iNOS expression in the cytoplasm of cardiomyocytes (black arrow). **D** The free liposome-treated group shows intense iNOS expression in the cytoplasm of cardiomyocytes (black arrow). **E** ACCF-liposomes treated group shows limited iNOS expression in the cytoplasm of cardiomyocytes. **F** The chart represents iNOS expression (area %) data presented as mean ± SEM. A significant difference is considered at *P* < 0.05

Figure 6B, D shows that the immunoperoxidase color reaction revealed higher iNOS activity in the hearts of untreated myocardial infarcted rats and those treated with free liposomes compared to the control group (Fig. 6A). The immunoreactive iNOS was mainly located in the cytoplasm of cardiomyocytes and vascular smooth muscle cells, with no presence in fibrotic areas (Fig. 6B, D). In contrast, myocardial infarcted rats treated with ACCF and its liposomal formulation had lower iNOS expression in the cytoplasm of cardiomyocytes, as shown in Fig. 6C, E. The ACCF-liposome-treated group also showed a significant (*P* < 0.05) decrease in iNOS-positive staining compared to the ACCF group, as seen in Fig. 6F.

### Effect of ACCF and ACCF-liposomes on Beclin-1 Expression

**Fig. 7:**
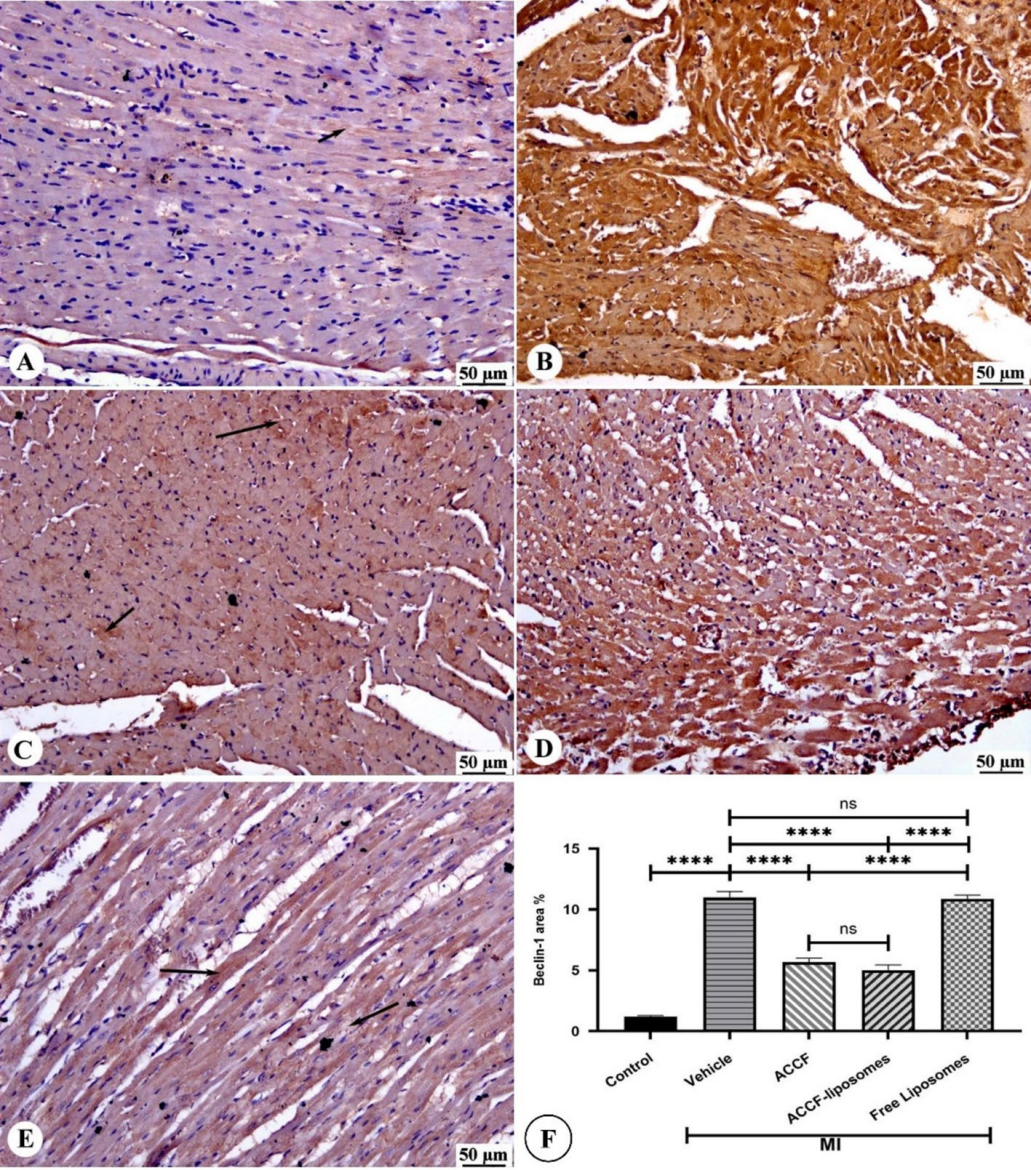
Photomicrograph of Beclin-1 immunostained heart sections. **A** The control group shows limited Beclin-1 expression in the cytoplasm of cardiomyocytes. **B** The myocardial infarcted group showed high positive cytoplasmic immunoreactivity (brown color), detected in most of the myofibers. **C** ACCF-treated group shows decreased Beclin-1 expression in the cytoplasm of cardiomyocytes (black arrow). **D** The free liposome-treated group shows intense Beclin-1 expression in the cytoplasm of cardiomyocytes (brown color). **E** ACCF-liposome-treated group shows decreased Beclin-1 expression in the cytoplasm of cardiomyocytes (black arrow). **F** The chart represents Beclin-1 expression (area %) data presented as mean ± SEM. A significant difference is considered at *P < 0*.05

The immunoperoxidase color reaction analysis revealed high cytoplasmic immunoreactivity of Beclin-1 in untreated rats with myocardial infarction and treated with free liposomes (Fig. 7B, D). This immunoreactivity was observed in most myofibers compared to the control group (Fig. 7A). However, myocardial infarcted rats treated with ACCF and ACCF-liposomes exhibited decreased Beclin-1 expression in the cytoplasm of cardiomyocytes (Fig. 7C, E). Additionally, morphometric analysis demonstrated a significant (*P* < 0.05) increase in Beclin-1 immunoreactivity in the myocardial infarcted group (Fig. 7F) compared to the control group. Conversely, myocardial infarcted rats treated with ACCF and ACCF-liposomes showed a significant (*P* < 0.05) reduction in Beclin-1 positive staining compared to the vehicle group, as depicted in Fig. 7 F.

### Comparison Between the Efficacies of ACCF and ACCF-liposomes in Attenuating Myocardial Infarction

**Fig. 8:**
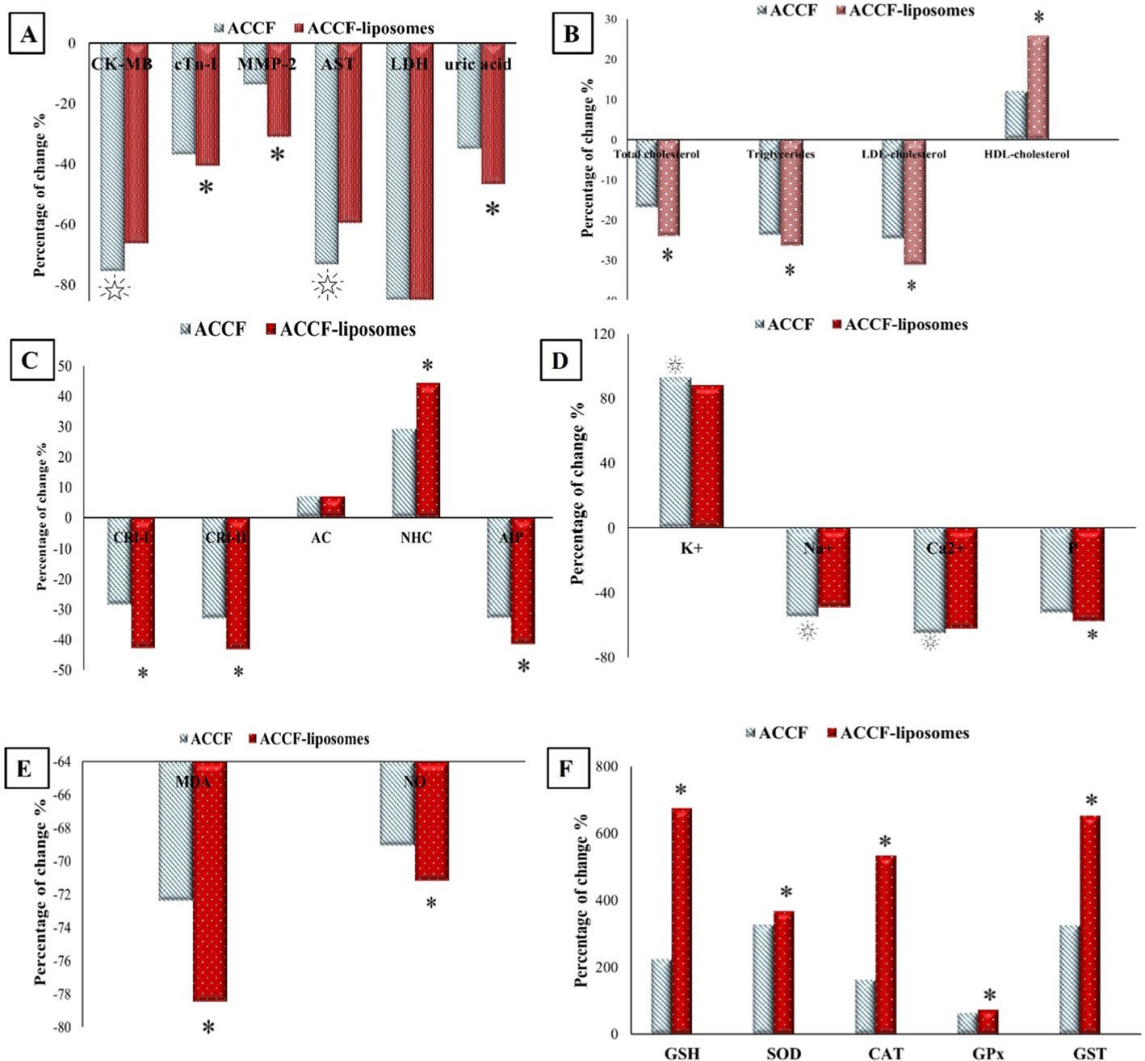
Comparison between the efficacies of ACCF and ACCF-liposomes in attenuating myocardial infarction. **A** Percentage change in cardiac markers of myocardial infarcted rats treated with ACCF and ACCF-liposomes. **B** Percentage change in lipid profile of myocardial infarcted rats treated with ACCF and ACCF-liposomes. **C** Percentage change in cardiovascular risk indices of myocardial infarcted rats treated with ACCF and ACCF-liposomes. **D** Percentage change in serum electrolytes of myocardial infarcted rats treated with ACCF and ACCF-liposomes. **E** Percentage change in cardiac oxidative markers of myocardial infarcted rats treated with ACCF and ACCF-liposomes. **F** Percentage change in cardiac antioxidant markers of myocardial infarcted rats treated with ACCF and ACCF-liposomes. * represents ACCF-liposomes as the most effective remedy;  represents ACCF as the most effective remedy

The results mentioned earlier show that both ACCF and ACCF-liposomes can partially reverse the changes induced by myocardial infarction. Using ACCF loaded on liposomes as a cardioprotective remedy is more effective than using ACCF alone because it can improve most altered myocardial infarction markers. The ACCF-liposomes treatment restored altered serum levels of cardiac function markers such as MMP-2, cTn-I, LDH, and uric acid more effectively than the untreated MI group (Fig. 8A). However, ACCF treatment improved some myocardial markers, such as serum Ck-MB and AST activities (Fig. 8A). ACCF-liposomes treatment was also the most effective in restoring the lipid profile and cardiovascular risk indices compared to the untreated MI group (Fig. 8B, C). On the other hand, ACCF treatment was better for preserving serum electrolyte levels (Fig. 8D). Furthermore, ACCF-liposomes treatment was the most effective in restoring the alteration in oxidative/antioxidative stress markers compared to the untreated myocardial infarcted rats (Fig. 8E, F). These results suggest that treatment with ACCF-liposomes may be more effective for myocardial infarction.

## Discussion

Inflammation and oxidative stress play a crucial role in the pathogenesis of myocardial infarction [32]. Consequently, there is a growing interest in exploring natural antioxidants as potential therapeutic agents for targeting these critical pathways. These antioxidants may offer the ability to modulate inflammation and oxidative stress, thereby enhancing cardiac functional recovery and improving survival rates of myocardial infarction (MI) [33]. *Allolobophora caliginosa*, an earthworm species, has garnered attention in complementary and alternative medicine (CAM) due to its abundant bioactive compounds, particularly the coelomic fluid. Recent studies have highlighted the potential of *Allolobophora caliginosa* in improving cardiac remodeling and aiding post-myocardial infarction (MI) recovery by scavenging reactive oxygen species (ROS) and alleviating inflammation, attributed to its coelomic fluid (ACCF) [9, 11]. Therefore, in the current investigation, a clinically relevant experimental approach utilizing liposomal delivery has been employed to augment the bioavailability and antioxidant effects of ACCF in managing the progression of myocardial infarction.

Excessive adrenaline worsens myocardial damage by increasing the heart’s oxygen demand, leading to weight loss due to a higher metabolic rate and fat breakdown. Thus, adrenaline effectively induces myocardial infarction, replacing dead muscle cells with scar tissue and increasing the heart and ventricular weight ratio in cases of myocardial infarction [34]. The findings indicate a significant (*P* < 0.05) increase in heart and ventricular weight ratio, alongside a reduction in body weight, in rats given an adrenaline overdose compared to the control group. This suggests cardiac hypertrophy occurs to maintain blood flow to remaining heart muscle cells, increasing the relative heart weight, as Tamis-Holland [35] stated. Moreover, a marked decline in overall body weight may be due to muscle hypertrophy, fluid retention, anxiety, and stress [36].

The study indicated that excess adrenaline resulted in significant ECG changes compared to the control group, consistent with previous studies [37, 38]. Increased QRS interval, P-wave duration, and ST-segment elevation may indicate ventricular functional abnormality due to oxidative stress [37]. This could potentially lead to membrane damage and an increased risk of ventricular arrhythmia and sudden cardiac collapse [39]. Specifically, an elevated ST-segment and P-wave duration is associated with complete blockage of the coronary artery and severe heart muscle injury. Additionally, it results in the shortening of the QT interval, attributed to an accelerated ventricular repolarization process, typically arising from partial coronary artery blockage. These changes may be due to excessive stimulation of beta-adrenergic receptors, which can damage the myocardium’s function and structure, interrupting membrane permeability and reliability [40].

In the present study, the development of cardiomyopathy in rats was indicated by elevated levels of CK-MB, cTn-I, MMP-2, AST, and LDH in the blood, recognized as key cardiac injury biomarkers. This physiological change was accompanied by significant histopathological alterations in the cardiac tissue, which aligns with previous research findings [41]. Disruption of cardiomyocyte membranes caused the release of CK-MB, AST, and LDH from the cytosol into circulation. This impairment likely results from excessive ROS and cytotoxic free radicals generated by adrenaline, damaging myocardial membranes and releasing intracellular cardiac enzymes [42]. A study conducted by Meng et al. [43] demonstrated that adrenaline binding to cardiac receptors activates pathways leading to cardiac hypertrophy, arrhythmias, and remodeling, ultimately resulting in myocardial cell death. This pathway breaks down glycogen into glucose for energy production, increasing metabolic activity and enhancing muscle contractility, releasing markers into the bloodstream [44]. Adrenaline administration increases calcium influx in cardiac myocytes, altering contractility and function. This results in a significant (*P* < 0.05) rise in CK-MB and cTn-I levels, as well as elevated AST and LDH activities in myocytes and blood [45]. These outcomes relate to a larger infarct area in myocytes, impaired cardiac function, and disrupted antioxidants and lipid peroxidation. Excessive adrenaline administration can trigger inflammation and tissue remodeling pathways, compromising the infarct’s integrity, causing cellular leakage and heart degeneration, ultimately leading to elevated myocardial markers, as Zhang et al. [46] reported.

The current study demonstrated a statistically significant (*P* < 0.05) increase in MMP-2 levels after adrenaline administration compared to the control group. The heightened MMP-2 activity has been linked to the progression of inflammation in infarcted areas. This observation implicates an intensified proteolytic activity within the systemic circulation, potentially leading to the degradation of contractile proteins and subsequently contributing to an augmented infarct size of the heart [47]. The elevated MMP-2 activity is attributed to the activation of ß-adrenergic receptors by adrenaline, as proposed by Rietz and Spiers [48].

Elevated uric acid after adrenaline increases platelet reactivity and inflammation while impairing endothelial function. The study suggests high uric acid and cardiac dysfunction may result from local angiotensin II receptor activation, cellular senescence, and oxidative stress [49]. Urate crystals from hyperuricemia are thought to cause inflammation and oxidative stress, contributing to cardiac parenchymal damage [50]. The elevated serum uric acid levels could be due to the activation of xanthine oxidase during hypoxic conditions. This enzyme catalyzes the conversion of hypoxanthine to xanthine and subsequently to uric acid and superoxide, leading to higher uric acid levels in the serum [51].

Hypercholesterolemia and hypertriglyceridemia are risk factors for atherosclerotic cardiovascular diseases, particularly coronary heart disease and myocardial infarction, impacting left ventricular function [52]. In this study, myocardial infarcted rats showed significantly higher total cholesterol, LDL, and TG levels, with lower HDL cholesterol than the control group. Excessive adrenaline can induce myocardial stress by depleting energy molecules like ATP and creatine phosphate in cardiomyocytes, potentially leading to myocardial infarction, as Olatunji et al. [53] stated. The ongoing study suggests that the heightened levels of cholesterol and triglycerides observed during myocardial infarction are ascribed to the activation of pro-inflammatory cytokines [54]. These cytokines stimulate hepatic density-lipoprotein production, increasing serum cholesterol and triglycerides [55].

Myocardial infarction is associated with an imbalance in serum electrolytes such as calcium, potassium, sodium, and phosphorus, which are attributed to ischemia [56]. This study found that administering adrenaline disrupted electrolyte balance. This disruption occurs due to inflammation, lipid buildup, and damage to the endothelium [57]. Rafaqat et al. [56] demonstrated that excessive adrenaline boosts aldosterone release, enhancing sodium and calcium reabsorption in the kidneys while promoting potassium excretion and reducing bloodstream potassium levels. The calcium influx can affect calcium channel blockers in myocardial cells, triggering events like generating reactive oxygen species, activating cell-death enzymes, and disrupting cellular energy and phosphorus due to coronary artery blockage. This constriction hinders blood flow to the heart muscle, resulting in insufficient oxygen supply to heart cells and damage [58]. Low potassium levels affect the heart’s electrical activity, disrupting impulses that regulate rhythm. This can compromise heart function and increase the risk of cardiovascular events [59].

Recent investigations have indicated that the overproduction of ROS resulting from inflammatory responses can contribute to myocardial infarction. This process can also cause degenerative changes in mitochondrial function, potentially leading to cardiac damage [45]. ROS can induce apoptotic cell death in cardiomyocytes, culminating in the deterioration of these cells and ultimately expediting infarction in cardiac cells, thereby resulting in damage and fibrosis [60]. This study linked cardiomyocyte damage to oxidative stress, marked by high malondialdehyde and nitric oxide levels, and a decrease in antioxidant capacity. Adrenaline triggers the sympathetic nervous system, boosting metabolism. As a result, this increased activity increases oxygen consumption and energy production. This can lead to higher reactive oxygen species (ROS) levels [61]. In addition, inflammation suppresses the immune response during myocardial infarction, thereby promoting lipid peroxidation and the production of MDA while reducing antioxidant levels in the cardiac tissue [62]. Demirci-Çekiç et al. reported that nitric oxide increases MMPs’ activity by stimulating pro-metalloproteinase production in cardiomyocytes [63]. Thus, the heightened MMP-2 activity in this study may result from excessive NO production in the heart tissue. The current study showed that adrenaline administration significantly reduced cardiomyocyte GSH content, possibly defending myocardial and endothelial cells from toxic oxidative and inflammatory environments following timely myocardial infarction compared to the control rats [64]. Tanzilli et al. suggested that reduced GSH levels could result from inflamed tissues excessively scavenging lipid peroxides [65]. This may promote infarct development and hinder T cell metabolic reprogramming and proliferation response.

Enzymatic antioxidant defense systems act as natural barriers against reactive oxygen species and lipid peroxidation, protecting cells from damage and scavenging free radicals [66]. This study found a marked reduction in the activities of antioxidant enzymes (CAT, SOD, GST, and GPx) in rats with myocardial infarction compared to controls. This reduced antioxidant enzyme activity likely stems from a lower capacity to neutralize superoxide and increased collagen deposition [67]. Lin et al. [68] disclosed that this decline may be related to changes in the left ventricle’s structure and increased cardiac fibrosis, which is closely tied to higher levels of oxidative stress in the blood vessels. According to Tan et al. [62], a decrease in antioxidant enzymes leads to the production of free radicals, causing damage to membrane lipids, compromising myocardial membrane permeability, and resulting in DNA fragmentation, causing various metabolic and morphological changes in cardiac tissues. DNA fragmentation is a potential indicator of tissue apoptosis, especially myocardium [69]. The current investigation clarified that excessive adrenaline-induced oxidative stress during myocardial infarction synergizes with DNA fragmentation, indicating cardiac apoptosis and necrosis. The observed DNA fragmentation in the heart muscle may be caused by excess ROS and reduced levels of antioxidants [70]. Furthermore, the DNA fragmentation might be a result of decreased SOD activity, leading to an accumulation of superoxide radicals. These radicals can then lead to the formation of hydroxyl radicals, which can, in turn, damage nearby DNA. This finding coincides with the results of Cowan [71]. Excessive production of nitric oxide because of iNOS activation may directly lead to the cleavage of DNA strands. This is corroborated by the observed upregulation in iNOS expression in myocardial infarcted rats compared to the control group. These findings align with Gao et al. [44], demonstrating that heightened iNOS activation during myocardial infarction stimulates nitric oxide production, initiating an inflammatory response that generates oxidative stress and inflammation. Consequently, this process leads to further tissue damage involving lipid peroxidation and DNA damage, exacerbating myocardial injury and dysfunction. Gough and Nolan [72] have documented that adrenaline induces iNOS expression in cardiac tissue through specific signaling pathways, including the cyclic adenosine monophosphate (cAMP) and prolonged sympathetic nervous system activation pathway. This produces excessive reactive oxygen species, such as peroxynitrite, in the infarcted myocardium.

Excessive intracellular reactive oxygen species or extracellular oxidative stress can lead to cellular death via the upregulation of autophagy [73]. ROS may function as a potent inducer of Beclin-1, a crucial mediator of autophagy during reperfusion. This study revealed an augmentation of autophagy in the myocardium after myocardial infarction, as evidenced by the heightened expression of Beclin-1 in infarcted myocardium of rats. This increase in autophagy may be attributed to an elevated level of calcium, which is a potent activator of autophagy [74]. These results are aligned with the previous studies of Chen et al. [75].

The study demonstrates that liposomal delivery of ACCF effectively reduces the severity of myocardial infarction. This is supported by significantly mitigating body weight loss, hypertrophic index, and ECG changes following treatment with ACCF-liposomes. The robust protective efficacy of ACCF-liposomes can be attributed to the presence of hydrophobic amino acid groups, particularly alanine and valine. These amino acids facilitate the scavenging of free radicals, thereby enabling targeted drug delivery to the infarction site. This process effectively reduces inflammation and limits damage [76]. Consequently, this reduces the size of the ventricular infarction and heart weight, accompanied by increased body weight. After myocardial infarction, ACCF and ACCF-liposomes significantly reduced (*P* < 0.05) myocardial markers, including cTn-I, CK-MB, MMP-2, AST, LDH, and uric acid content. These outcomes are associated with a smaller infarct area within the myocytes, improved cardiac function, and restored endogenous antioxidants. These results are consistent with Zhang et al. [46]. The cardioprotective effect of ACCF or its nano-liposomal formulation may be ascribed to its lysine content. Lysine is recognized for its antimicrobial properties, which can mitigate inflammation, hinder gelatin degradation, and types IV and V collagen. Consequently, it may contribute to the regulation of myocardial function. Furthermore, the antioxidant potential of ACCF-liposomes, attributed to their phenolic compounds, may facilitate the restoration of myocardial integrity, thereby reducing the release of myocardial enzymes into the bloodstream. This interpretation is corroborated by Dajem et al. [25]. This study found that nanoliposomes can effectively regulate cardiac biomarkers due to their size and adaptable properties, allowing for both active and passive targeting of cardiac tissues [77].

The current findings suggest that the administration of ACCF and its liposomal formulation improved the lipid profile in rats suffering from myocardial infarction. This intervention was also associated with reduced atherogenic indices and increased markers linked to cardiomyocyte protection. The efficacy of ACCF is attributed to its glycolipoprotein content, which includes insulin-like growth factor and epidermal growth factor, which are involved in regulating glucose and lipid metabolism. Consequently, this intervention appears to modulate lipid synthesis, transport, breakdown, and maintenance, thereby impacting the lipid profile and atherogenic indices in the treated rats [41]. Tan et al. [78] reported that liposomes reduce lipid metabolism by activating inflammasomes and macrophages and crystallizing cholesterol. These processes expand volume through plaque destruction or rupture. Thus, this study attributes the hypo-lipidemic potency of ACCF-liposomes to their ability to stabilize plaques, prevent macrophage intrusion, and inhibit foam cell formation by enhancing cholesterol outflow and suppressing inflammation responses, as disclosed by Luo et al. [79].

After a heart attack, treatment with ACCF and its liposomal formulation significantly reduced sodium, calcium, and phosphorus levels. This suggests that ACCF may help to protect the heart muscle and maintain the body’s electrolyte balance through its aspartic acid content, which supports the heart’s electrical function and osmoregulation, as supported by Dewi and Mahendra [80]. Moreover, according to Sadek et al. [13], the phenolic compounds of ACCF have been identified as the contributing factors to their antioxidant activity, which protects the kidneys from damage. This is particularly significant due to the kidneys’ crucial role in filtering and regulating electrolyte levels in the bloodstream. Additionally, the formulation of ACCF with nanoliposomes can be tailored to achieve sustained or controlled release of bioactive compounds over an extended duration. This controlled release profile has the potential to enhance therapeutic effects by ensuring consistent delivery of bioactive components to specific tissues and by more effectively regulating electrolyte levels. This was demonstrated in a prior study, which found that the nano-liposomal formulation resulted in sustained release of ACCF for 8 h [22]. According to Ekhlasian et al. [81], using liposome-encapsulated natural components helps regulate electrolyte balance by standardizing fluid absorption and discharge in the gastrointestinal system. This process contributes to maintaining electrolyte levels. Furthermore, it can help preserve the stability of mitochondrial transmembrane potential in heart and kidney cells, impacting mitochondrial metabolism and electrolyte balance, as Yang et al. [82] reported.

This study postulated that the cardioprotective potential of ACCF and ACCF-liposomes might be attributed to their capacity to chelate and scavenge free radicals, decrease oxidative damage, restore redox balance, and inhibit myocardial oxidative stress by preventing ROS generation. Since the antioxidant ability could form a stabilized phenoxy radical that can effectively scavenge excess free radicals and counteract cardiomyocyte damage. Furthermore, the antioxidant properties of ACCF can safeguard cells from oxidative damage by reducing lipid hydroperoxide produced during peroxidation, thus protecting cell structure by neutralizing free radicals, as Tan et al. [62] reported. This assumption is supported by their ability to reduce myocardial oxidative stress by significantly increasing myocardial GSH content and antioxidant enzyme activities (SOD, CAT, GPx, and GST), as well as markedly decreasing MDA and NO formation. These findings agree with the previous studies [83–85]. The high antioxidant potency of these substances can be attributed to their polyphenol and amino acid contents. These compounds can scavenge free radicals by donating hydrogen atoms from their phenolic hydroxyl groups [12, 25]. Additionally, they serve as direct precursors for synthesizing antioxidant enzymes [75]. The present study stated that the coelomic fluid possesses antioxidant properties due to its bioactive compounds, along with a high content of fatty acids such as hexadecenoic acid, octadecenoic acid, and decanoic acid, which have been shown to have antioxidant properties [12]. Moreover, Lazzarotto-Figueiró et al. [86] found the antioxidant activity of coelomic fluid due to its high omega-6, omega-9, phytosterols, and total flavonoids. This suggests it may reduce cholesterol and help prevent cardiovascular diseases by directly scavenging free radicals by donating a hydrogen atom and maintaining endogenous antioxidant enzymes. One of the promising strategies for counteracting the detrimental effects of free radical products in the heart and ameliorating the associated destructive changes is the utilization of a liposomal formulation of ACCF as an antiradical mediator. This is attributed to the small size and enhanced biological activity of ACCF, which contribute to its efficacy in neutralizing free radicals and mitigating their aggressive impacts. This protective mechanism operates by selectively targeting inflammatory sites and mitigating the production of cytokines and chemokines, thereby ameliorating heart injury [87]. Additionally, this approach leads to an increase in the activities of antioxidant enzymes while concurrently reducing levels of free radicals.

**Fig. 9:**
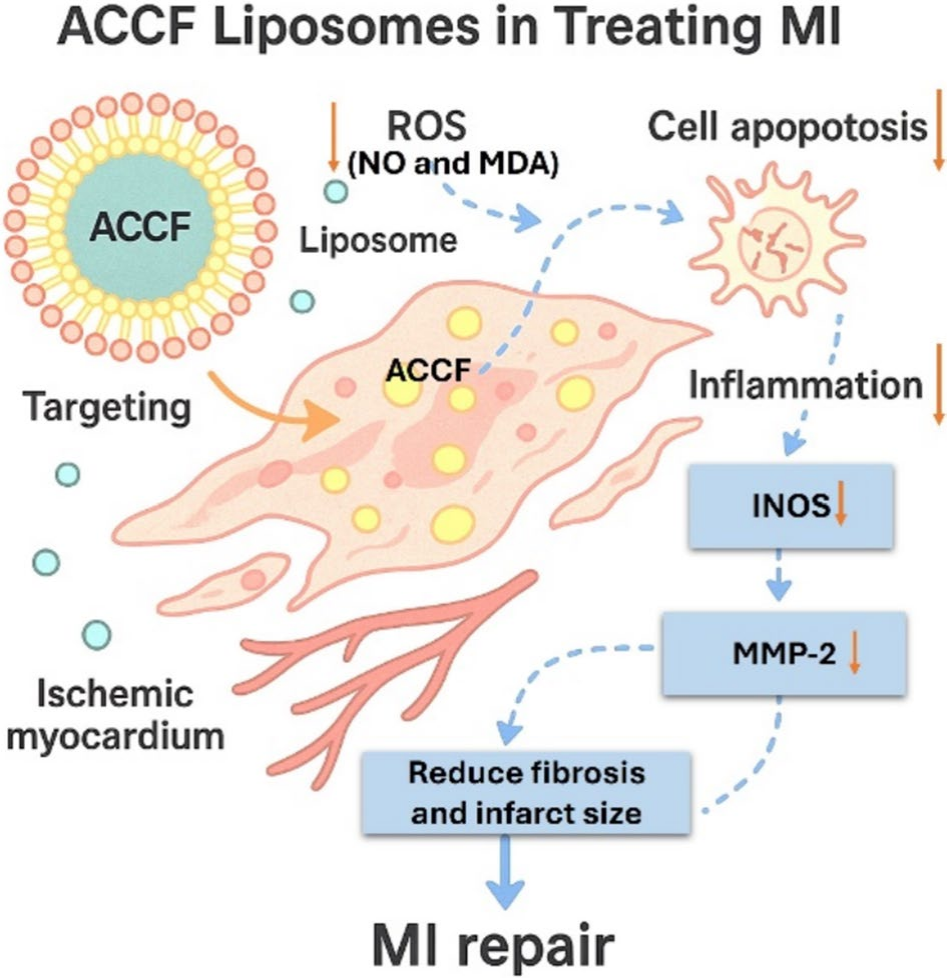
A representative diagram showing the molecular pathway for the liposomal delivery of ACCF in treating myocardial infarction

The current investigation has demonstrated that ACCF and ACCF-liposomes, as natural constituents possessing significant antioxidant, antiapoptotic, and anti-inflammatory attributes, exhibit the potential to attenuate DNA fragmentation in rats with myocardial infarction (Fig. 9). Notably, the anti-inflammatory efficacy of nanoliposomal formulations enables them to counteract inflammatory responses effectively. Consequently, this mechanism mitigates DNA mutation and fragmentation by preserving DNA integrity in myocardial infarcted rats, as evidenced by the findings of Shariare et al. [88]. Additionally, ACCF contains phenolic and flavonoid compounds, specifically gallic acid and quercetin [13]. These compounds exhibit effective free radical scavenging activity. ACCF also contains peptides with anti-apoptotic properties, reducing DNA fragmentation in myocardial infarcted rats. Also, ACCF and ACCF-liposomes treatment caused a marked decline in iNOS and Beclin 1 expression in myocardial infarcted rats. These findings were corroborated by the prior research conducted by Sadek et al. [13]. Their studies highlighted that coelomic fluid is endowed with anti-inflammatory properties, characterized by antimicrobial peptides, lectins, and cytokines. These bioactive compounds have exhibited immunomodulatory effects capable of regulating inflammatory responses, thereby potentially influencing iNOS activity and the level of Beclin 1. The reduction in iNOS expression may be attributed to the antioxidant properties of ACCF, which can scavenge free radicals and mitigate oxidative damage. Consequently, this may influence nitric oxide levels and subsequent signaling pathways. As a result, it may influence iNOS activity, NO availability, and cardiomyocyte infarction. Furthermore, the fluid may also play a role in modulating cellular stress responses, particularly those associated with autophagy regulation, and reducing the level of Beclin-1. This interpretation is supported by Manna et al. [89]. These findings were confirmed by improvements in the heart’s tissue structure. The study attributed the protective effects of ACCF on the heart’s structure to its bioactive compounds that have anti-inflammatory and immunomodulatory properties. These compounds have specific effects on the inflammatory response and tissue remodeling, which help restore the changes in the heart tissue [22].

## Conclusions

The investigation demonstrated that excessive adrenaline induces oxidative damage in cardiac tissue by initiating and propagating multiple reactive oxygen species. This process triggers inflammation, DNA fragmentation, and apoptosis, impacting the results of biochemical, histopathological, and immunohistochemical analyses. Notably, the study revealed that the liposomal delivery of ACCF ameliorates adrenaline-associated pathological changes, including attenuating oxidative damage, inflammation, and apoptosis. Furthermore, ACCF-liposomes downregulate the expression of iNOS and Beclin-1. Thus, the study suggested that ACCF-liposomes could serve as a prospective cardioprotective treatment to mitigate the repercussions of myocardial infarction. Although administering ACCF-liposomes to rats with myocardial infarction resulted in significant outcomes, further studies are needed to elucidate the exact molecular mechanism underlying the cardioprotective potential of ACCF.

## Data Availability

The datasets used and/or analyzed during the current study are available from the corresponding author on reasonable request.
